# Supplementary material for: SEM-2/SoxC regulates multiple aspects of C. elegans postembryonic mesoderm development
Source: bioRxiv. 2024 Jul 4:2024.07.04.602042. Preprint. [Version 1] doi: 10.1101/2024.07.04.602042 (PMC11245110; doi:10.1101/2024.07.04.602042)
Supplement: Supplement 1 [file NIHPP2024.07.04.602042v1-supplement-1.pdf]

bioRxiv preprint doi: https://doi.org/10.1101/2024.07.04.602042; this version posted July 4, 2024. The copyright holder for this preprint (which was not certified by peer review) is the author/funder, who has granted bioRxiv a license to display the preprint in perpetuity. It is made available under aCC-BY 4.0 International license.

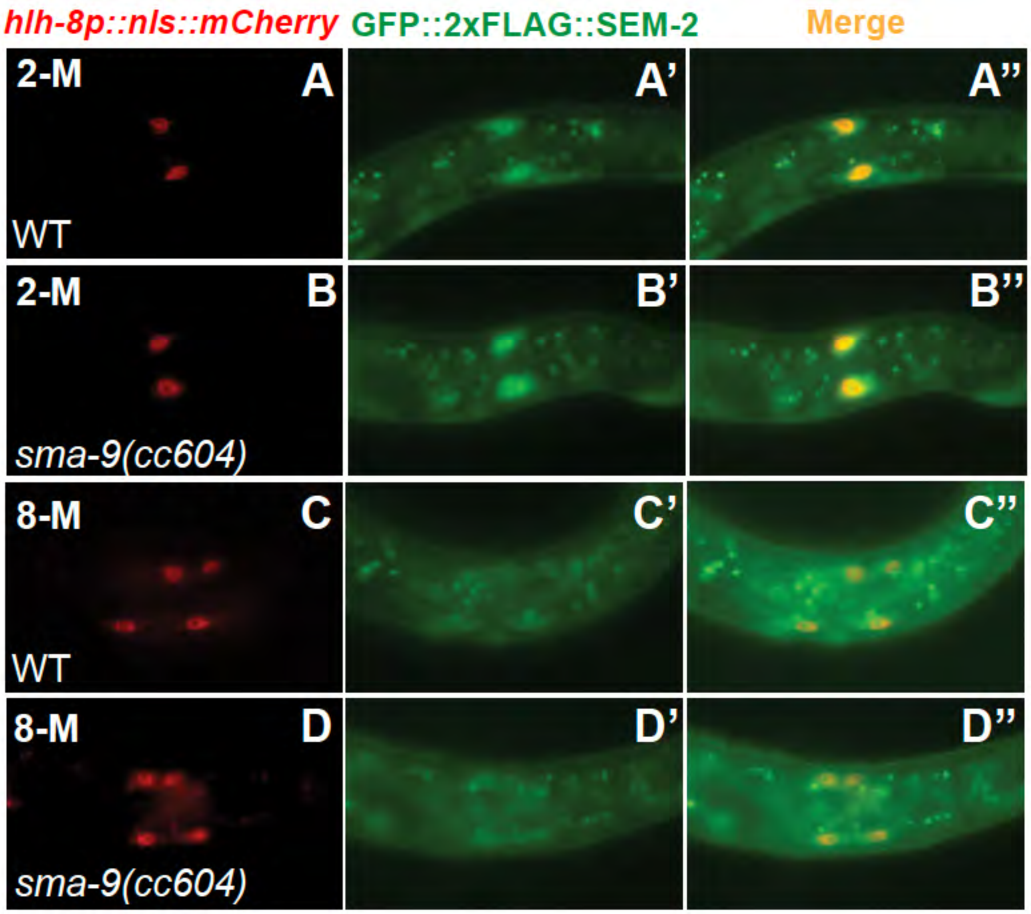

Supplementary Figure 1: *sem-2* expression in the early M lineage does not change in *sma-9(0)* mutants.

A–D") Florescence images showing GFP::2xFLAG::SEM-2 (A'–D') in M lineage cells labelled by the *hlh-8p::nls::mCherry* reporter (A–D) at the 2-M stage (A–B'') and 8-M stage (C–D'') of M lineage development in WT (A–A'', C–C") and *sma-9(0)* (B–B'', D–D'') hermaphrodites. (A''–D'') are the corresponding merged images. Only the left side of an animal is shown in this figure, while the other side is out of the focal plane.

bioRxiv preprint doi: https://doi.org/10.1101/2024.07.04.602042; this version posted July 4, 2024. The copyright holder for this preprint (which was not certified by peer review) is the author/funder, who has granted bioRxiv a license to display the preprint in perpetuity. It is made available under aCC-BY 4.0 International license.

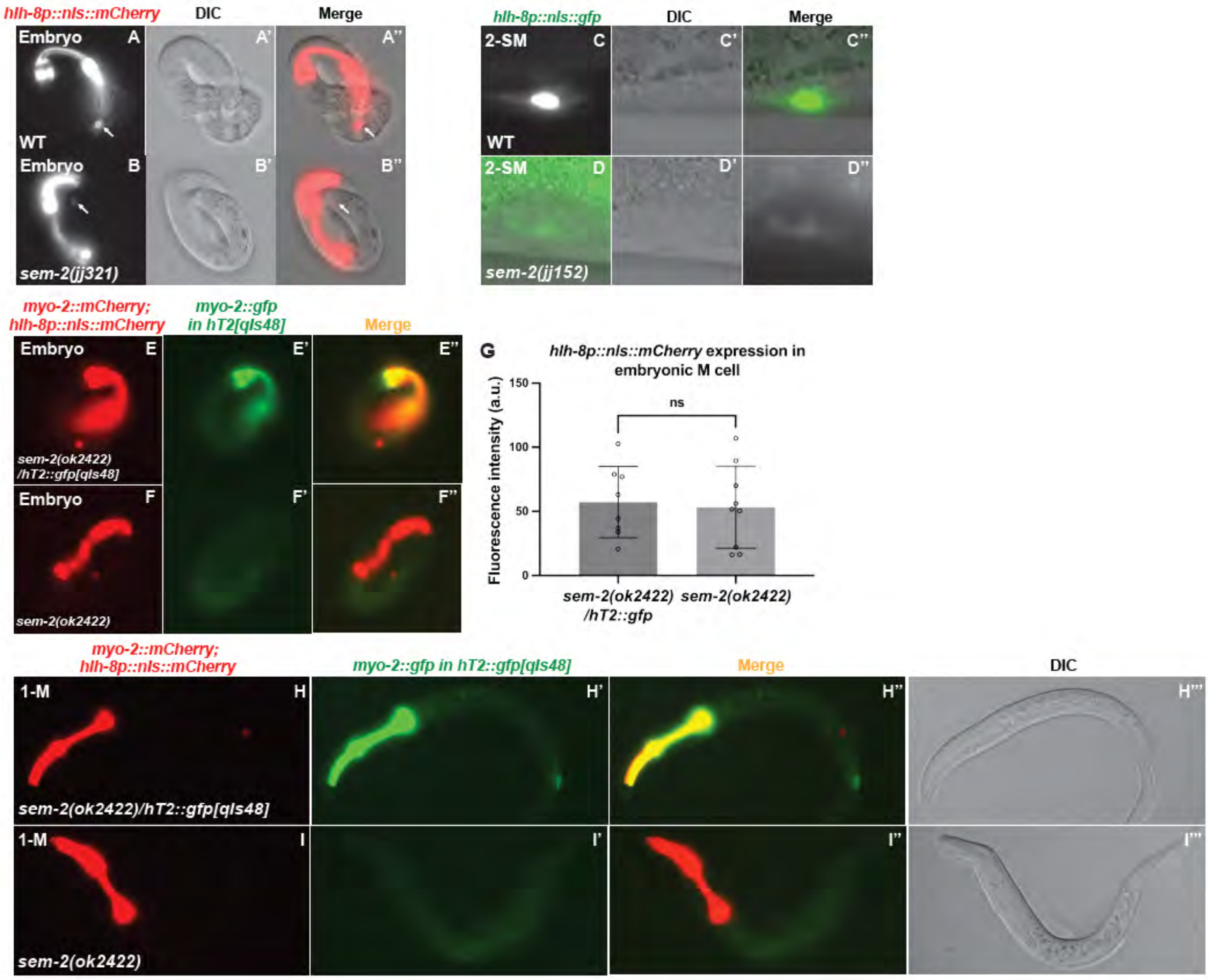

Supplementary Figure 2: The regulation of *hlh-8* by SEM-2 is not transgene- or *sem-2* allele-specific

A–B'') Fluorescence images (A–B) of wild-type (A–A'') and *sem-2(jj321[P158S])* (B–B'') embryos showing the expression of *hlh-8p::nls::mCherry* in the M mesoblast cell (arrows). A'–B' and A''–B'' are corresponding DIC and merged images, respectively. Transgenic animals expressing *hlh-8p::nls::mCherry* were generated with a co-injection marker *myo-2p::mCherry*. C–D'') Fluorescence images (C–D) of wild-type (C–C'') and *sem-2(jj152[P158S])* (D–D'') mutant L3 animals showing expression of *hlh-8p::gfp* in SMs. C'–D' and C''–D'' are corresponding DIC and merged images, respectively. Exposure for panel D is 20x times higher than panel C. E–G) Fluorescence images (E–F) of a heterozygous *sem-2* null (*sem-2(ok2422)/hT2::gfp[qIs48]*) (E–E'') and a *sem-2* null *(sem-2(ok2422))* (F–F'') embryo showing expression of *hlh-8p::nls::mCherry*. *hT2::gfp[qIs48]* fluorescence images are shown in E'–F' and merged images are shown in E''–F''. G) Quantification of *hlh-8p::nls::mCherry* expression in the M cell of *sem-2(ok2422)/hT2::gfp[qIs48]* and *sem-2(ok2422)* animals. Each dot represents an animal scored.

bioRxiv preprint doi: https://doi.org/10.1101/2024.07.04.602042; this version posted July 4, 2024. The copyright holder for this preprint (which was not certified by peer review) is the author/funder, who has granted bioRxiv a license to display the preprint in perpetuity. It is made available under aCC-BY 4.0 International license.

Statistical significance was calculated by performing unpaired two-tailed Student's t-tests. ns, not significant. H–I''') Fluorescence images (H–I) of a heterozygous *sem-2* null (*sem-2(ok2422)/hT2::gfp[qIs48]*) (H–H''') and a *sem-2* null *(sem-2(ok2422))* (I–I''') L1 animals showing expression of *hlh-8p::nls::mCherry*. *hT2::gfp[qIs48]* images are shown in H'–I', merged images of *hlh-8p::nls::mCherry* and *hT2::gfp[qIs48]* are shown in H''–I'', and DIC images are shown in H'''–I'''.

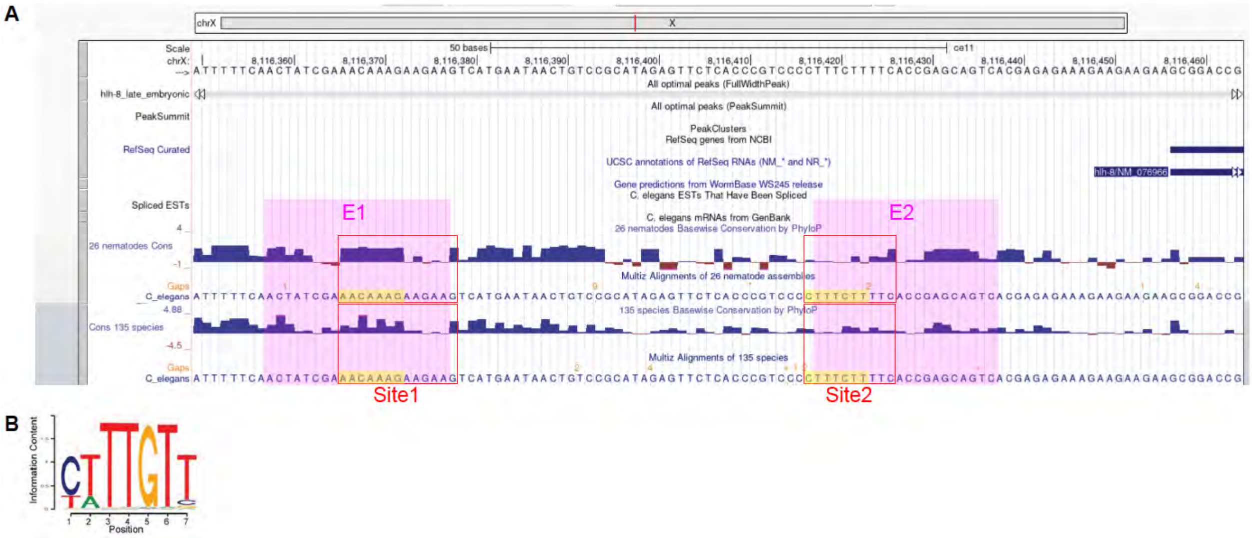

Supplementary Figure 3: The putative SEM-2/SoxC-binding site in E1 is conserved.

A) Screenshot of the University of California, Santa Cruz genome browser showing the sequence conservation of E1 and E2 in the *hlh-8* promoter among 26 nematode species and a broader 135 species (112 nematodes, 22 flatworms, and *Ciona intestinalis)*. The magenta color highlights E1 and E2. Red boxes show Site1 and Site2. The yellow color highlights the putative SEM-2/SoxC-binding sites in E1/Site1 and E2/Site2. B) The position weight matrix of the Sox4/SoxC primary motif as determined by the protein-binding microarray (PBM) method (Jauch et al. 2012).

bioRxiv preprint doi: https://doi.org/10.1101/2024.07.04.602042; this version posted July 4, 2024. The copyright holder for this preprint (which was not certified by peer review) is the author/funder, who has granted bioRxiv a license to display the preprint in perpetuity. It is made available under aCC-BY 4.0 International license.

Supplementary table 1. *C. elegans* strains used in this study

Strain ID	Genotype
**Strains carrying the SEM-2 P158S mutation**	
LW5780	*sem-2(jj152) I*
LW5914	*sem-2(jj320) I*
LW5915	*sem-2(jj321) I*
**Strains carrying other *sem-2* mutations**	
LW1926	*hT2[qIs48] (I;III)/sem-2(ok2422) I*
LW1221	*sem-2(n1343) I*
LW2734	*sem-2(n1343) I; ccIs4438(intrinsic CC:::gfp) III; ayIs2(egl-15p::gfp) IV; sma-9(cc604) ayIs6(hlh-8p::gfp) X*
LW3880	*sem-2(n1343) I; ccIs4438(intrinsic CC:::gfp) III; ayIs2(egl-15p::gfp) IV; ayIs6(hlh-8p::gfp) X*
**Strains carrying endogenously-tagged SEM-2**	
LW6287	*sem-2(jj382[gfp::2xflag::sem-2]) I*
LW6282	*sem-2(jj382[gfp::2xflag::sem-2]) I; jjIs3900* [*hlh-8p::nls::mCherry::lacZ+myo-2::mCherry] IV*
LW6466	*sem-2(jj382[gfp::2xflag::sem-2]) I; jjIs3900* [*hlh-8p::nls::mCherry::lacZ+myo-2::mCherry] IV; sma-9(cc604) X*
LW6467	*sem-2(jj382 jj417[gfp::2xflag::sem-2 P158S] I*
LW6537	*sem-2(jj382 jj417[gfp::2xflag::sem-2 P158S] I; jjIs3900* [*hlh-8p::nls::mCherry::lacZ+myo-2::mCherry] IV*
**Strains carrying various reporters**	
LW3949	*jjIs3900* [*hlh-8p::nls::mCherry::lacZ+myo-2::mCherry] IV*
LW0081	*ccIs4438(intrinsic CC:::gfp) III; ayIs2(egl-15p::gfp) IV; ayIs6(hlh-8p::gfp) X*
SYS668	*let-381(dev205[mNeonGreen::let-381]) I*
**Strains used for examining the Susm phenotype of the SEM-2 P158S mutation**	
LW2598	*arIs37[secreted CC::gfp] I; cup-5(ar465) III; sma-9(cc604) X*
LW5924	*sem-2(jj152) arIs37[secreted CC::gfp] I; cup-5(ar465) III; sma-9(cc604) X*
LW5903	*sem-2(jj152) arIs37[secreted CC::gfp] I; cup-5(ar465) III; sma-9(cc604) X; jjIs1647[N-GFP::sem-2 fosmid + pRF4]*
**Strains for examining the M lineage phenotypes in SEM-2 P158S mutants**	
LW6928	*let-381(dev205[mNeonGreen::let-381]) I; jjIs3900 [hlh-8p::nls::mCherry::lacZ+myo-2::mCherry] IV*
LW6727	*sem-2(jj476[SEM-2 P158S]) let-381(dev205[mNeonGreen::let-381] I; jjIs3900 [hlh-8p::nls::mCherry::lacZ+myo-2::mCherry] IV*
LW6929	*let-381(dev205[mNeonGreen::let-381]) I; jjIs3900 [hlh-8p::nls::mCherry::lacZ+myo-2::mCherry] IV; sma-9(cc604) X*
LW6934	*sem-2(jj476[SEM-2 P158S]) let-381(dev205[mNeonGreen::let-381]) I; jjIs3900 [hlh-8p::nls::mCherry::lacZ+myo-2::mCherry] IV; sma-9(cc604) X isolate #1*

bioRxiv preprint doi: https://doi.org/10.1101/2024.07.04.602042; this version posted July 4, 2024. The copyright holder for this preprint (which was not certified by peer review) is the author/funder, who has granted bioRxiv a license to display the preprint in perpetuity. It is made available under aCC-BY 4.0 International license.

LW6935	*sem-2(jj476[SEM-2 P158S]) let-381(dev205[mNeonGreen::let-381]) I; jjIs3900 [hlh-8p::nls::mCherry::lacZ+myo-2::mCherry] IV; sma-9(cc604) X isolate #2*
LW5847	*sem-2(jj152) I; ccIs4438[intrinsic CC::gfp] III isolate #1*
LW5848	*sem-2(jj152) I; ccIs4438[intrinsic CC::gfp] III isolate #2*
LW5845	*sem-2(jj152) I; ccIs4438[intrinsic CC::gfp] III; arIs2[egl-15::gfp] IV isolate #1*
LW5846	*sem-2(jj152) I; ccIs4438[intrinsic CC::gfp] III; arIs2[egl-15::gfp] IV isolate #2*
**Strains with transgenic *hlh-8* promoter deletions**	
LW0649	*jjEx649[pAYL11(hlh-8p(517bp)::gfp) + PRF4] line #1*
LW0652	*jjEx652[pAYL11(hlh-8p(517bp)::gfp) + PRF4] line #2*
LW0793	*jjEx793[pAYL11(hlh-8p(517bp)::gfp) + PRF4] line #3*
LW0787	*jjEx787[pAYL21(hlh-8p(deletion)::gfp + PRF4] line #1*
LW0790	*jjEx790[pAYL21(hlh-8p(deletion)::gfp + PRF4] line #2*
LW0366	*jjEx366[pAYL21(hlh-8p(deletion)::gfp + PRF4] line #3*
LW0798	*jjEx798[pAYL22(hlh-8p(deletion)::gfp + PRF4] line #1*
LW0802	*jjEx802[pAYL22(hlh-8p(deletion)::gfp + PRF4] line #2*
LW0651	*jjEx651[pAYL23(hlh-8p(deletion)::gfp + PRF4] line #1*
LW0788	*jjEx788[pAYL23(hlh-8p(deletion)::gfp + PRF4] line #2*
LW0801	*jjEx801[pAYL23(hlh-8p(deletion)::gfp + PRF4] line #3*
LW0796	*jjEx796[pAYL24(hlh-8p(deletion)::gfp + PRF4] line #1*
LW0803	*jjEx803[pAYL24(hlh-8p(deletion)::gfp + PRF4] line #2*
LW0808	*jjEx808[pAYL24(hlh-8p(deletion)::gfp + PRF4] line #3*
LW0811	*jjEx811[pAYL25(hlh-8p(deletion)::gfp + PRF4] line #1*
LW0800	*jjEx800[pAYL31(hlh-8p(deletion)::gfp + PRF4] line #1*
LW0806	*jjEx806[pAYL31(hlh-8p(deletion)::gfp + PRF4] line #2*
LW0785	*jjEx785[pAYL32(hlh-8p(deletion)::gfp + PRF4] line #1*
LW0791	*jjEx791[pAYL32(hlh-8p(deletion)::gfp + PRF4] line #2*
LW0792	*jjEx792[pAYL32(hlh-8p(deletion)::gfp + PRF4] line #3*
LW0795	*jjEx795[pAYL32(hlh-8p(deletion)::gfp + PRF4] line #4*
LW0789	*jjEx789[pAYL33(hlh-8p(deletion)::gfp + PRF4] line #1*
LW0794	*jjEx794[pAYL33(hlh-8p(deletion)::gfp + PRF4] line #2*
LW0786	*jjEx786[pAYL35(hlh-8p(deletion)::gfp + PRF4] line #1*

bioRxiv preprint doi: https://doi.org/10.1101/2024.07.04.602042; this version posted July 4, 2024. The copyright holder for this preprint (which was not certified by peer review) is the author/funder, who has granted bioRxiv a license to display the preprint in perpetuity. It is made available under aCC-BY 4.0 International license.

LW0804	*jjEx804[pAYL35(hlh-8p(deletion)::gfp + PRF4] line #2*
LW0805	*jjEx805[pAYL35(hlh-8p(deletion)::gfp + PRF4] line #3*
LW6717	*jjEx6717[pAYL11(hlh-8p(517bp)::gfp) + pJKL449(myo-2p::gfp)]; jjIs3900 [hlh-8p::nls::mCherry::lacZ + myo-2::mCherry] IV*
LW6720	*jjEx6720[pAYL35(hlh-8p(deletion)::gfp) + pJKL449(myo-2p::gfp)]; jjIs3900 [hlh-8p::nls::mCherry::lacZ + myo-2::mCherry] IV line #1*
LW6751	*Ex[pAYL35(hlh-8p(deletion)::gfp) + pJKL449(myo-2p::gfp)]; jjIs3900 [hlh-8p::nls::mCherry::lacZ + myo-2::mCherry] IV line #2*
LW6718	*jjEx6718[pAYL32(hlh-8p(deletion)::gfp) + pJKL449(myo-2p::gfp)]; jjIs3900 [hlh-8p::nls::mCherry::lacZ + myo-2::mCherry] IV line #1*
LW6719	*jjEx6719[pAYL32(hlh-8p(deletion)::gfp) + pJKL449(myo-2p::gfp)]; jjIs3900 [hlh-8p::nls::mCherry::lacZ + myo-2::mCherry] IV line #2*
**Strains with the endogenous *hlh-8* transcriptional reporter**	
LW6459	*hlh-8(jj422[hlh-8p::hlh-8::sl2::nls::gfp::nls::hlh-8 3' UTR]) X*
LW6476	*jjIs3900 [hlh-8p::nls::mCherry::lacZ+myo-2::mCherry] IV; hlh-8(jj422[hlh-8p::hlh-8::sl2::nls::gfp::nls::hlh-8 3' UTR]) X isolate #1*
LW6477	*jjIs3900 [hlh-8p::nls::mCherry::lacZ+myo-2::mCherry] IV; hlh-8(jj422[hlh-8p::hlh-8::sl2::nls::gfp::nls::hlh-8 3' UTR]) X isolate #2*
LW6478	*sem-2(jj321) I; jjIs3900 [hlh-8p::nls::mCherry::lacZ+myo-2::mCherry] IV; hlh-8(j422[hlh-8p::hlh-8::sl2::nls::gfp::nls::hlh-8 3' UTR]) X isolate #1*
LW6479	*sem-2(jj321) I; jjIs3900 [hlh-8p::nls::mCherry::lacZ+myo-2::mCherry] IV; hlh-8(j422[hlh-8p::hlh-8::sl2::nls::gfp::nls::hlh-8 3' UTR]) X isolate #2*
**Strains with endogenous *hlh-8* promoter mutations**	
LW6498	*hlh-8(jj445 jj422[hlh-8p(13bp mutation at -272bp to -259bp in the hlh-8 promoter)::hlh-8::sl2::nls::gfp::nls::hlh-8 3' UTR]) X*
LW6499	*hlh-8(jj446 jj422[hlh-8p(13bp mutation at -272bp to -259bp in the hlh-8 promoter)::hlh-8::sl2::nls::gfp::nls::hlh-8 3' UTR] X*
LW6686	*hlh-8(jj483 jj422[hlh-8p(10bp mutation at -221bp to -211bp and 13bp mutation at -272bp to -259bp in the hlh-8 promoter)::hlh-8::sl2::nls::gfp::nls::hlh-8 3' UTR] X*
LW6528	*jjIs3900 [hlh-8p::nls::mCherry::lacZ + myo-2::mCherry] IV; hlh-8(jj445 jj422[hlh-8p(13bp mutation at -272bp to -259bp in the hlh-8 promoter)::hlh-8::sl2::nls::gfp::nls::hlh-8 3' UTR]) X isolate #1*
LW6529	*jjIs3900 [hlh-8p::nls::mCherry::lacZ + myo-2::mCherry] IV; hlh-8(jj445 jj422[hlh-8p13bp mutation at at -272bp to -259bp in the hlh-8 promoter)::hlh-8::sl2::nls::gfp::nls::hlh-8 3' UTR]) X isolate #2*
LW6822	*jjIs3900 [hlh-8p::nls::mCherry::lacZ + myo-2::mCherry] IV; hlh-8(jj446 jj422[hlh-8p(13bp mutation at at -272bp to -259bp in the hlh-8 promoter)::hlh-8::sl2::nls::gfp::nls::hlh-8 3' UTR] X isolate #1*
LW6699	*jjIs3900 [hlh-8p::nls::mCherry::lacZ + myo-2::mCherry] IV; hlh-8(jj483 jj422[hlh-8p(10bp mutation at at -221bp to -211bp and 13bp mutation at -272bp to -259bp in the hlh-8 promoter)::hlh-8::sl2::nls::gfp::nls::hlh-8 3' UTR] X isolate #1*
LW6700	*jjIs3900 [hlh-8p::nls::mCherry::lacZ + myo-2::mCherry] IV; hlh-8(jj483 jj422[hlh-8p(10bp mutation at -221bp to -211bp and 13bp mutation at -272bp to -259bp in the hlh-8 promoter)::hlh-8::sl2::nls::gfp::nls::hlh-8 3' UTR] X isolate #2*
**Strains for examining the M lineage phenotypes in SEM-2 P158S mutants**	
LW5845	*sem-2(jj152) I; ccIs4438[intrinsic CC::gfp] III; arIs2[egl-15p::gfp] IV isolate #1*
LW5846	*sem-2(jj152) I; ccIs4438[intrinsic CC::gfp] III; arIs2[egl-15p::gfp] IV isolate #2*
LW5847	*sem-2(jj152) I; ccIs4438[intrinsic CC::gfp] III isolate #1*
LW5848	*sem-2(jj152) I; ccIs4438[intrinsic CC::gfp] III isolate #2*
LW5931	*sem-2(jj152) I; ayIs2(egl-15p::gfp) IV; ayIs6(hlh-8p::gfp) X isolate #1*
LW5932	*sem-2(jj152) I; ayIs2(egl-15p::gfp) IV; ayIs6(hlh-8p::gfp) X isolate #2*

bioRxiv preprint doi: https://doi.org/10.1101/2024.07.04.602042; this version posted July 4, 2024. The copyright holder for this preprint (which was not certified by peer review) is the author/funder, who has granted bioRxiv a license to display the preprint in perpetuity. It is made available under aCC-BY 4.0 International license.

LW6474	*sem-2(jj321) I; jjIs3900 [hlh-8p::nls::mCherry::lacZ+myo-2::mCherry] IV isolate #1*
LW6475	*sem-2(jj321) I; jjIs3900 [hlh-8p::nls::mCherry::lacZ+myo-2::mCherry] IV isolate #2*
LW4072	*jjIs3644[egl-15p::rfp(pJKL737)+dpy-20(+)] II; hlh-29::gfp [TLM908] III*
LW5996	*sem-2(jj321) I; jjIs3644[egl-15p::rfp(pJKL737)+dpy-20(+)] II; hlh-29p::gfp [TLM908] III isolate #1*
LW5997	*sem-2(jj321) I; jjIs3644[egl-15p::rfp(pJKL737)+dpy-20(+)] II; hlh-29p::gfp [TLM908] III isolate #2*
LW5998	*sem-2(jj321) I; jjIs3644[egl-15p::rfp(pJKL737)+dpy-20(+)] II; hlh-29p::gfp [TLM908] III isolate #3*
LW3189	*vsIs4[rgs-2p::gfp]*
LW6473	*sem-2(jj321) I; vsIs4[rgs-2p::gfp]*
LW3430	*ccIs4443[arg-1p::gfp] IV*
LW6385	*sem-2(jj321) I; ccIs4443[arg-1p::gfp] IV isolate #1*
LW6386	*sem-2(jj321) I; ccIs4443[arg-1p::gfp] IV isolate #2*
LW1379	*jjIs1379[pJKL743(NdEbox::nls::gfp+unc-119(+)]; unc-119(ed4) III*
LW6415	*sem-2(jj321) I; jjIs1379[pJKL743(NdEbox::nls::gfp+unc-119(+)]*
IX4506	*mls-2(vy248[mNeonGreen::mls-2]) X*
LW6684	*sem-2(jj321) I; mls-2(vy248[mNeonGreen::mls-2]) X isolate #1*
LW6685	*sem-2(jj321) I; mls-2(vy248[mNeonGreen::mls-2]) X isolate #2*

bioRxiv preprint doi: https://doi.org/10.1101/2024.07.04.602042; this version posted July 4, 2024. The copyright holder for this preprint (which was not certified by peer review) is the author/funder, who has granted bioRxiv a license to display the preprint in perpetuity. It is made available under aCC-BY 4.0 International license.

Supplementary table 2. Oligonucleotides used in this study

Oligo ID	Sequence
**Repair oligo for generating SEM-2 P158S**	
MDB-21	For generating *sem-2(jj320)*, *sem-2(jj321), sem-2(jj417) and sem-2(jj476)*<br><br>AGGAAATACTGTAACCGGATTTTTCGAATAATTGTATTGTAATTTTAAATTTTTTCAGGAATACTCAGATTACAA GTACAAGCCACGTAAAAAGCCGAAAAAGAACCCAGATGGAACACTTCAGCAGCCAGCTCAACCCCAAGCTC
**Repair oligos for generating mutations in the *hlh-8* promoter**	
MDB-97	For generating a 13bp mutation *at* -272bp to -259bp in the *hlh-8* promoter [*hlh-8(jj445) and hlh-8(jj446)]*<br><br>TTCTACCTTCACTCTCAAATTCTTTTTTCAGCGGTAATTTTTCAACTATCGAGGCGCCGCAGGCCTCATGAATA ACTGTCAGCATAGAGTTCTCACCCGTCCCCTTTCTTTTCACCGAG
MDB-146	For generating a 10bp mutation at -221bp to -211bp and a 13bp mutation *at* -272bp to -259bp in the *hlh-8* promoter [*hlh-8(jj483)]*<br><br>GTGTTAGTGTAGGTTGCTTTGCTTCTACCTTCACTCTCAAATTCTTTTTTCAGCGGTAATTTTTCAACTATCGA GGCGCCGCAGGCCTCATGAATAACTGTCAGCATAGAGTTCTCACCCGTCCCCCGAGGCCGGACCGAGCAG TCACGAGAGAAAGAAGAAGAAGCGGACCGCTGCAGAGATTCTTCGTAGCGG
**sgRNA for CRISPR**	
sgRNA-MDB-1	guide #1 targeting the *hlh-8* promoter to generate *hlh-8(jj445), hlh-8(jj446),* and *hlh-8(jj483)*<br><br>CGATAGTTGAAAAATTACCG
sgRNA-MDB-2	guide #2 targeting the *hlh-8* promoter to generate *hlh-8(jj445), hlh-8(jj446),* and *hlh-8(jj483)*<br><br>GGACGGGTGAGAACTCTATG
sgRNA-MDB-7	guide #1 targeting *sem-2* to generate *sem-2(jj476)*<br><br>TTCTTCTTTGGCTTCTTGCG
sgRNA-MDB-8	guide #2 targeting *sem-2* to generate *sem-2(jj476)*<br><br>CGTGGCTTGTATTTGTAGTC
**For genotyping *sem-2(jj152)* and *sem-2(jj320/1)* i.e. SEM-2 P158S**	
JKL-1910	GTCAGTGTAGGAGGTAGGTG
JKL-1911	GGCATCTTTTGTGCATGACTC
**For amplification to genotype for *sem-2(jj152)* and *sem-2(jj320/1)* i.e. SEM-2 P158S**	
JKL-1910	GTCAGTGTAGGAGGTAGGTG
JKL-1911	GGCATCTTTTGTGCATGACTC
**For genotyping *sem-2(ok2422)***	
CXT-46	CAGATATCAAATGGATCTCC
CXT-47	GTCCTTTTGACAGCTTATCAC
CXT-48	CAATGCATCGCTCCATGGATAA
**For genotyping *sem-2(jj382)***	
MDB-P15 (for screening and homozygosing)	ACGAGGATTGGGACAACTCC

bioRxiv preprint doi: https://doi.org/10.1101/2024.07.04.602042; this version posted July 4, 2024. The copyright holder for this preprint (which was not certified by peer review) is the author/funder, who has granted bioRxiv a license to display the preprint in perpetuity. It is made available under aCC-BY 4.0 International license.

JKL-1922 (for screening)	CTCGAGAGAGAGAGAGAGAAATG
MDB-65 (for homozygosing)	CTGCCGAAATTCGGTCTCCTG
CXT-47 (for homozygosing)	GTCCTTTTGACAGCTTATCAC
**For genotyping *hlh-8(jj380/1)***	
MDB-56	GAAATCATGCTGAACTATGG
MDB-57	TTCGAACCACGCGTTCTCC
MDB-58	ATTCATGACTTCTTCTAAGC
**For genotyping *hlh-8(jj445/6)***	
MDB-56	GAAATCATGCTGAACTATGG
MDB-57	TTCGAACCACGCGTTCTCC
MDB-99	TTTCAACTATCGAGGCGCC
**For genotyping *hlh-8(jj483)***	
MDB-56	GAAATCATGCTGAACTATGG
MDB-57	TTCGAACCACGCGTTCTCC
MDB-145	GACTGCTCGGTCCGGCCTCGG
**For genotyping *hlh-8(jj422)***	
MDB-89 (for screening)	GTCACTAGTCTTGGATGTCAGTACC
MDB-90 (for screening)	GAAGAAGCGTAAGGTACCGG
MDB-84 (for homozygosing)	AGAAGAATTTCATGAACTGTGTCCC
MDB-85 (for homozygosing)	CAATGGGCTTACTAATAGTCTCTTGC
MDB-86 (for homozygosing)	GGAGAACTTGTGTCCGTTGAC

bioRxiv preprint doi: https://doi.org/10.1101/2024.07.04.602042; this version posted July 4, 2024. The copyright holder for this preprint (which was not certified by peer review) is the author/funder, who has granted bioRxiv a license to display the preprint in perpetuity. It is made available under aCC-BY 4.0 International license.

Supplementary table 3. Plasmids generated in this study.

Plasmid ID	Details
***hlh-8* promoter deletion constructs**	
pAYL11	*517bp(-517* to -1*) hlh-8p::gfp::unc-54 3' UTR*
pAYL21	*517bp(deletion of -401 to -350) hlh-8p::gfp::unc-54 3' UTR*
pAYL22	*517bp(deletion of -351 to -300) hlh-8p::gfp::unc-54 3' UTR*
pAYL23	*517bp(deletion of -301 to -250) hlh-8p::gfp::unc-54 3' UTR*
pAYL24	*517bp(deletion of -251 to -200) hlh-8p::gfp::unc-54 3' UTR*
pAYL25	*517bp(deletion of -201 to -150) hlh-8p::gfp::unc-54 3' UTR*
pAYL31	*517bp(deletion of -300 to -281) hlh-8p::gfp::unc-54 3' UTR*
pAYL32	*517bp(deletion of -280 to -261) hlh-8p::gfp::unc-54 3' UTR*
pAYL33	*517bp(deletion of -260 to -241) hlh-8p::gfp::unc-54 3' UTR*
pAYL34	*517bp(deletion of -240 to -221) hlh-8p::gfp::unc-54 3' UTR*
pAYL35	*517bp(deletion of -220 to -201) hlh-8p::gfp::unc-54 3' UTR*
**Plasmids used for CRISPR**	
pMDB15	sgRNA plasmid #1 for making the SEM-2 P158S mutation [*sem-2(jj321), sem-2(jj320), and sem-2(jj417)] (in pRB1017)*<br>MDB-19: TCTTGATTCTTCTTTGGCTTCTTGCG<br>MDB-20: AAACCGCAAGAAGCCAAAGAAGAATC
pVG1	sgRNA plasmid #2 for making the SEM-2 P158S mutation [*sem-2(jj321), sem-2(jj320), and sem-2(jj417)] (in pRB1017)*<br>JKL-1912: TCTTGCGTGGCTTGTATTTGTAGTC<br>JKL-1913: AAACGACTACAAATACAAGCCACGC
pMDB27	sgRNA plasmid #1 for generating *gfp::2xflag::sem-2 [sem-2(jj382)] (in pRB1017)*<br>MDB-32: TCTTGAATCGGCGGCATCATATGC<br>MDB-33: AAACGCATATGATGCCGCCGATTC
pMDB29	sgRNA plasmid #2 for generating *gfp::2xflag::sem-2 [sem-2(jj382)] (in pRB1017)*<br>MDB-44: TCTTGCCAACTTCATGCTGGATTG<br>MDB-45: AAACCAATCCAGCATGAAGTTGGC
pMDB28	*Repair template for generating gfp::2xflag::sem-2 [sem-2(jj382)]*
pMDB33	sgRNA plasmid #1 for generating the endogenous *hlh-8* transcriptional reporter *hlh-8(jj422[hlh-8p::hlh-8::sl2::nls::gfp::nls::hlh-8 3'UTR])* (in *pRB1017*)<br>MDB-69: TCTTGGAATTGGGATTTGGAGTTGAGA<br>MDB-70: AAACTCTCAACTCCAAATCCCAATTCC
pMDB34	sgRNA plasmid #2 for generating the endogenous *hlh-8* transcriptional reporter *hlh-8(jj422[hlh-8p::hlh-8::sl2::nls::gfp::nls::hlh-8 3'UTR])* (in *pRB1017*)<br>MDB-71: TCTTGTGGAGTGAGTTGATTTGGATT<br>MDB-72: AAACAATCCAAATCAACTCACTCCAC
pMDB35	sgRNA plasmid #3 for generating the endogenous *hlh-8* transcriptional reporter *hlh-8(jj422[hlh-8p::hlh-8::sl2::nls::gfp::nls::hlh-8 3'UTR])* (in *pRB1017*)

bioRxiv preprint doi: https://doi.org/10.1101/2024.07.04.602042; this version posted July 4, 2024. The copyright holder for this preprint (which was not certified by peer review) is the author/funder, who has granted bioRxiv a license to display the preprint in perpetuity. It is made available under aCC-BY 4.0 International license.

	MDB-73: TCTTGTCCAAATCAACTCACTCCAATTCAC<br>MDB-74: AAACGTGAATTGGAGTGAGTTGATTTGGAC
pMDB36	sgRNA plasmid #4 for generating the endogenous *hlh-8* transcriptional reporter *hlh-8(jj42[hlh-8p::hlh-8::sl2::nls::gfp::nls::hlh-8 3'UTR])* (in *pRB1017*)<br>MDB-75: TCTTGTTATTGCCAGTGAATTGGAG<br>MDB-76: AAACCTCCAATTCACTGGCAATAAC
pMDB37	*Repair template for generating hlh-8(jj422[hlh-8p::hlh-8::sl2::nls::gfp::nls::hlh-8 3'UTR])*

All plasmids were verified by Sanger sequencing.
